# Parliament and the Profession

**Published:** 1916-10-21

**Authors:** 


### Parliament and the Profession.

### Examining Medical Officers.

In answer to a question by Captain Bennett-Goldney as to the reason for the large variation in the remuneration of examining medical officers, the Financial Secretary to the War Office explained that the 24s. rate per day is appropriate to continuous employment, while a maximum of 40s. is payable for occasional service only.

### Import of Salicylates.

The President of the Board of Trade was asked what weight and what value of salicylates were imported into the United Kingdom in the years 1911-1915, and what proportion came from Germany. In answer it was stated that analysis of these imports entailed much labour, but the following figures, taken from the actual declarations of importers in 1913, could be quoted: Salicylic acid, total imports £107,031, from Germany £10,375; sodium salicylate, total £16,147, German £15,264; acetyl salicylate, total £3,518, German £3,376; aspirin, total £20,096, German £19,974; the only quantity that could be quoted was for sodium salicylate, of which 2,385 cwt. were declared, including 2,305 cwt. from Germany.

### Sale of Cocaine.

Unregistered dental practitioners have had the exemption under which they may purchase solutions containing 0.1 per cent. or more of cocaine extended until October 31, after which date, Mr. Herbert Samuel declared himself as satisfied, other efficient local anæsthetics will be available in sufficient quantities. This reply was in answer to Mr. Hogge, who in response to a further inquiry elicited the information that it was not proposed to apply the restrictions to registered dentists.

### Chlorine Gas Experiments.

In answer to a question put to the Home Secretary by Mr. Chancellor, Mr. Roberts stated that the eighty-four experiments with chlorine gas on cats and other animals, reported in the Return for 1915 of the Medical Research Committee, were performed by a member of the staff of that Committee, which was established under the National Insurance Act, and that the cost of the work was defrayed from moneys provided by Parliament under the Act.

